# The Management of the Aortic Arch in Type A Aortic Dissection: Replace, Repair with the AMDS, or Leave for Another Day?

**DOI:** 10.3390/jcdd12010023

**Published:** 2025-01-12

**Authors:** Ryaan EL-Andari, Michael C. Moon

**Affiliations:** Division of Cardiac Surgery, Department of Surgery, University of Alberta, Edmonton, AB T6G 2R3, Canada; elandari@ualberta.ca

**Keywords:** aortic dissection, aortic arch, AMDS hybrid prosthesis

## Abstract

Objectives: Acute type A aortic dissection (ATAAD) is a life-threatening condition that requires emergent surgical intervention. Numerous surgical approaches exist for ATAAD, and controversy remains regarding the optimal arch interventions for ATAAD patients. Aortic Arch Interventions: Approaches to ATAAD repair include hemiarch repair or extended arch repairs, including the hemiarch with a hybrid stent implantation, such as the AMDS hybrid Prosthesis, total arch replacement (TAR), and the use of an elephant trunk and frozen elephant trunk. While indications for each procedure exist, such as entry tears in the arch, arch aneurysms, and head vessel communications for TAR and malperfusion and a reduced risk of distal anastomotic new entry tears in Debakey I aortic dissection for the AMDS and frozen elephant trunks, the optimal intervention depends on numerous factors. Surgeon and center experience, resource availability, patient risk, and anatomy all contribute to the decision-making process. TAR has improved in safety over the years and has been demonstrated to be comparable to the hemiarch repair in terms of safety in many settings. TAR may also prevent adverse remodeling and can effectively treat more distal diseases, the presence of arch tears, arch aneurysms, and branch vessel involvement or malperfusion. Conclusions: Numerous surgical approaches exist to manage ATAAD, allowing for the surgeon to tailor the repair to the individual patient and pathology. TAR allows for single or staged repair of extensive pathologies and can address distal entry tears, the aneurysmal arch, and head vessel pathologies. In cases with malperfusion, an AMDS can be used in many cases. The management strategy for ATAAD should always involve performing the best surgery for the patient, although in cases where a total arch is indicated but cannot be performed safely by a non-aortic surgeon, the safest approach may be to perform a hemiarch initially and to plan for an elective arch reoperation in the case it is required following close surveillance.

## 1. Introduction

Acute type A aortic dissection (ATAAD) is a surgical emergency. With high rates of adverse outcomes, early surgical intervention is required to reduce the risk of morbidity and mortality [1,2,3]. Despite early intervention and large volumes of data published in the literature regarding the management of ATAAD patients, several points regarding surgical approach remain uncertain.

The hemiarch repair is the minimum required intervention to prevent death and to stabilize the aortic arch. Even in patients with dissection extending into and beyond the arch, a hemiarch repair is often sufficient as an initial intervention [4]. Widely debated are the indications for aortic arch interventions in addition to the selection of aortic arch intervention to pursue. While a hemiarch replacement is often a life-saving procedure, the presence of several conditions such as an aneurysmal arch, re-entry tears, connective tissue disease, and malperfusion all warrant further consideration when determining the operative plan for a patient presenting with ATAAD [2,3,5,6]. Malperfusion is of significant concern, as it has been associated with mortality, stroke, heart failure, renal failure, spinal cord injury, limb ischemia, elevated lactate, and required reintervention [4,7,8].

Further complicating this matter are the questions of short-term risk versus long-term benefit and the experience of the surgeon. While an aortic arch intervention may provide benefits in terms of malperfusion or reintervention, there is a considerable increase in operative time and potential risk with extending an operation into the aortic arch [3,9,10]. Furthermore, while all cardiac surgeons are expected to be able to perform a hemiarch repair, this is not the case for aortic arch interventions. Aortic arch interventions can be complex and are often performed by trained aortic surgeons. While aortic surgeons are available at many large cardiac care centers, aortic surgeons are not available at all centers and may not be on call every day unless a dedicated aortic call is established. As ATAAD is an emergent and time-sensitive condition, aortic surgeons may not be available for all ATAAD cases. Given the complex decision-making required when evaluating a patient for surgical repair of ATAAD, we aim to review the important considerations related to aortic arch interventions for patients presenting with ATAAD.

## 2. Technical Considerations

### 2.1. Hemiarch Repair

Prior to the initiation of the aortic repair, there are several considerations for setup of the case from the anesthesiology perspective. Monitoring the temperature, neuromonitoring, and monitoring blood pressure are key for the case. The placement of arterial lines proximally, such as in the right radial, and distally, such as in the femoral arteries or left radial, will help to determine true blood pressure following repair and reperfusion. Transesophageal echocardiography is essential for monitoring intraoperatively. Following reperfusion and rewarming, communication with the anesthesiology team is paramount to obtaining hemostasis, as input from both laboratory values and the surgical team regarding hemostasis will guide the administration of blood products.

**Figure 1:**
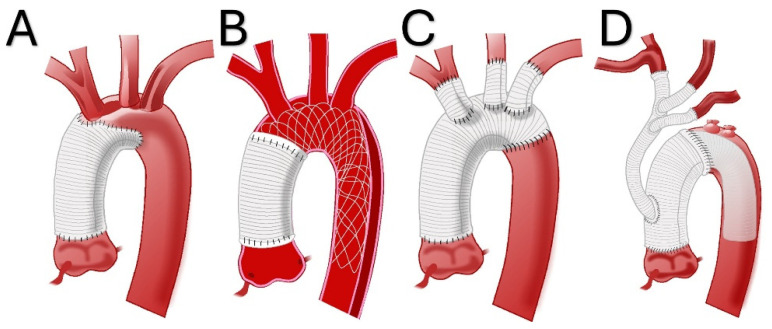
Illustrations of various repairs for acute type A aortic dissection, including hemiarch repair (**A**), repair with the AMDS hybrid prosthesis (**B**), total arch repair (**C**), and total arch repair with a frozen elephant trunk and debranching to zone 0 (**D**).

**Figure 2:**
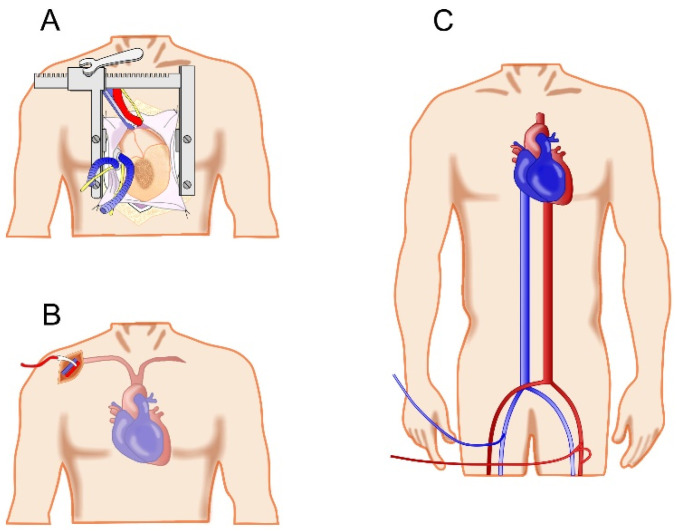
Illustrations of cannulation strategies for cardiopulmonary bypass, including central cannulation (**A**), right axillary arterial cannulation (**B**), and femoral cannulation (**C**).

A hemiarch repair initially involves cannulation for cardiopulmonary bypass (CPB) (Figure 1A). Several approaches are utilized depending on patient anatomy and surgeon experience. In contrast to central cannulation (Figure 2A), peripheral cannulation allows for the rapid establishment of CPB in the case of cardiac arrest or aortic rupture. Peripheral venous access is often achieved through femoral venous cannulation. Arterial cannulation can be performed through an axillary cutdown or through femoral arterial access [1,9,11,12] (Figure 2B,C). Axillary arterial cannulation is often preferred, as it provides several advantages, including cerebral perfusion following circulatory arrest and clamping of the innominate artery without additional cannulation of the head vessels, and is associated with a reduced risk of embolism to the brain compared to the retrograde flow associated with femoral cannulation [9,11,12]. Central cannulation of the aorta and the right atrium are also utilized in certain cases [9,12,13]. Central aortic cannulation requires care, and echocardiographic guidance allows for visualization of the placement of a wire in the true lumen to prevent cannulation of the false lumen [9]. Alternate approaches to cannulation that may be utilized in emergent situations include direct true lumen cannulation (Samurai technique), and direct LV apical cannulation may be considered to allow for antegrade flow and reduced organ malperfusion [14,15]. While some previous studies have found comparable outcomes between cannulation strategies, the results of previous meta-analyses have found lower rates of mortality and cerebrovascular accidents with axillary cannulation [9]. Once CPB is established, cooling is commenced, and the aortic repair may begin.

The hemiarch repair involves a proximal anastomosis in either the ascending aorta or aortic root, depending on patient presentation, and a distal anastomosis in zone 0 at the proximal portion of the aortic arch. Given the location of the distal anastomosis is in the aortic arch and must be performed with the cross-clamp off, this can be started once cooling has been achieved to the target temperature and circulatory arrest is started. The target temperature varies based on several factors, including the extent of required repair, predicted circulatory arrest time, and surgeon preference, but are most commonly between 18 and 26 °C. Lower temperatures allow for greater cerebral, spinal, and visceral organ protection but are associated with longer warming periods and worsening coagulopathy. Once the patient’s circulation has been arrested, cerebral perfusion is recommended, especially for longer cases and where the circulatory arrest temperature is higher. Cerebral perfusion may be performed with either antegrade cerebral perfusion through the axillary artery, the direct cannulation of the carotid arteries, or retrograde perfusion through the superior vena cava [12,16,17,18].

The distal anastomosis is prepared by resecting the aorta to zone 0 and beveling the inferior aspect of the aortic arch. A graft is then prepared, and the distal anastomosis is performed. Once the distal anastomosis is performed, the graft can be clamped and circulation re-established.

### 2.2. AMDS Hybrid Prosthesis

The AMDS hybrid prosthesis (Artivion, GA, USA) is a hybrid stent device used in ATAAD. The AMDS comprises a helical stent design and a proximal sewing cuff (Figure 1B). The AMDS has been demonstrated to improve aortic remodeling following ATAAD repair and has shown the most benefit in patients with signs of malperfusion.

The AMDS is deployed during a hemiarch repair. The general preparation and setup are similar to the hemiarch approach as described above. At the distal anastomosis site, the aortotomy is kept proximal to the innominate artery and is transverse, as opposed to the standard bevel for the hemiarch. Approximately 1 cm of aorta proximal to the innominate artery is left for a sewing cuff. The transverse aortotomy is preferred over the angled bevel for AMDS insertion, as the proximal sewing cuff of the AMDS is circular and smaller than the distal aorta if it were to be beveled. A transverse aortotomy allows for an easier anastomosis that is more closely approximated between the cuff and aorta. The AMDS is brought into the field, and the distal end is placed inside the aortic arch into the descending thoracic aorta. The AMDS is then deployed. The proximal cuff is then incorporated into the distal anastomosis.

In the original Dissected Aorta Repair Through Stent (DARTS) Implantation trial, the deployment of the AMDS was not found to add significant time to the surgical procedure. Furthermore, the deployment of the AMDS is relatively simple and can easily be performed by any cardiac surgeon during a hemiarch repair.

### 2.3. Total Arch Repair

Aortic arch interventions usually require a similar setup to a hemiarch repair in terms of cannulation and circulatory arrest. An aortic arch repair encompasses a wide range of interventions, and the use of an elephant trunk (ET) or frozen elephant trunk (FET) have been increasing [19,20,21,22] (Figure 1C,D). The simplest arch intervention requires resection of the aortic arch and replacement with a graft. The proximal end of this anastomosis in ATAAD is most commonly in the ascending aorta or aortic root. The distal end of this anastomosis will depend on the additional procedures performed and the anatomy of the dissection. The distal end of this anastomosis may be anywhere from zone 0–3, with isolated arch replacements often being anastomosed more distally.

The head vessels may be managed in several different ways. For perfusion of the head vessels, unilateral antegrade cerebral perfusion is often sufficient. In cases of malperfusion of the head vessels, this may not be sufficient to perfuse the brain, resulting in a drop in cerebral oxygen saturations. In these cases, bilateral antegrade cerebral perfusion or retrograde cerebral perfusion may provide optimal cerebral blood flow. Retrograde cerebral perfusion also allows for retrograde flushing of any potential thrombus or debris from the cerebral circulation and carotid arteries if present. The head vessels are most commonly anastomosed individually to the aortic graft, either to a branched graft or to interposition grafts, which are then anastomosed to the main body of the arch graft. In order to facilitate moving the distal anastomosis proximally, debranching of the arch and anastomosis of the head vessels more proximally allows for a distal anastomosis between zones 0 and 2 and the use of antegrade stents to complete a FET without concern of covering the head vessels [4,17,19,23]. The more proximal anastomosis in zones 0–2 combined with an arch device such as a FET have several benefits while still treating the entire arch, including a less challenging distal anastomosis, easier control of bleeding at the distal suture line should it arise, and a reduced risk of laryngeal nerve injury.

There may be cases where extensive dissection of the head vessels, poor head vessel tissue quality, or distal thrombus may complicate head vessel reconstruction. Several approaches may be utilized in these cases. Where extensive head vessel dissection or poor tissue quality is present, resection of the head vessels back to better quality tissue may be feasible, although this may require an interposition graft in some cases. Reconstruction of the adventitia with autologous pericardium may be useful as well to reinforce the anastomosis. In cases where reconstruction of the head vessels in the chest is not possible due to extensive dissection or poor tissue quality, ligation of the vessel and an extra-anatomic bypass may be required to restore perfusion. As previously mentioned, if thrombus is suspected to be present in the distal carotid arteries, retrograde cerebral perfusion may help to flush remaining thrombus from the carotid arteries. Finally, in cases where following the reconstruction and restoration of continuity of antegrade blood flow there continues to be obstruction or thrombus present, a postoperative referral to interventional radiology for thrombectomy or head vessel stenting may be considered.

At the distal anastomosis, several options exist. The graft may be anastomosed directly to the distal aortic arch. A conventional ET may be utilized, which is a free piece of fabric at the distal end of the arch graft that will sit within the lumen of the aorta and is used to facilitate future endovascular interventions, providing a landing zone for such devices or surgical repair by providing a clamp site and sufficient cuff to suture to during a thoracoabdominal aortic repair. A FET describes the use of either an endovascular covered stent deployed in the distal arch and descending thoracic aorta or a hybrid graft with both an arch graft and distal [17,19,20,24,25]. This device may be deployed antegrade at the distal anastomosis or may be performed as a staged intervention in a retrograde fashion through the femoral arteries as a standard endovascular aortic repair (EVAR) using the arch graft as a proximal landing zone.

More recently, hybrid grafts have been utilized that include a proximal aortic graft for the arch anastomosis and an antegrade EVAR within one device [4,22,23]. Some examples include the Thoraflex (Terumo Aortic, Scotland, United Kingdom), E-vita (Artivion, GA, USA), and Frozenix (Japan Lifeline, Tokyo, Japan) grafts [19,22,23,26,27,28]. These are various dacron grafts that have the antegrade FET portion supported by a nitinol stent. The distal end is deployed into the arch or descending thoracic aorta and is sewn to the distal anastomosis. The arch can then be reconstructed onto the proximal portion of the dacron graft [19,22,23,26,27,28]. Endovascular arch grafts also exist. These include the Medtronic Valiant left subclavian artery branched device, the Cook arch branched stent graft, the Endospan Nexus graft, and the Terumo Relay branch graft [23]. These devices vary in their deployment, with the Valiant graft being deployed in zone 2 with a branch that is deployed in the left subclavian artery. Several arch grafts exist with two branches, such as the Terumo relay, which requires revascularization of the left subclavian artery [23]. While these devices benefit from avoiding open surgery, their use has been limited to high-risk cases and are not frequently used compared to open arch repairs in the setting of ATAAD.

Variations of this technique have also been described, such as the branched stented anastomosis frozen elephant trunk repair (B-SAFER) technique. This technique also has several variations but generally involves the deployment of a covered stent graft into the descending thoracic aorta, following which a fenestration is created and a stent graft is deployed into one (the left subclavian artery) or more arch vessels through the graft. The proximal portion of the graft is anastomosed to a combination of the deployed graft and remaining aortic arch tissue in an extended hemiarch fashion [29]. These allow for a total arch repair with complete revascularization of the head vessels without debranching the subclavian artery or performing an extra-anatomic bypass to the vessel.

## 3. Current Approaches to the Aortic Arch

### 3.1. Hemiarch Repair

As mentioned previously, the hemiarch repair is the standard intervention for any ATAAD. The goal of the hemiarch repair is to resect the primary entry tear, stabilize the aortic arch, and prevent complications, such as aortic rupture or tamponade [4]. In the case of Debakey Type II dissection, this will be contained in the ascending aorta. In the case of Debakey I dissection, the dissection will extend into the arch and descending thoracic aorta, although a hemiarch repair may still be adequate as an initial intervention. The hemiarch repair is taught to all cardiac surgeons and can be performed at any center with cardiac surgery. The hemiarch repair is acceptable for the vast majority of ATAAD patients, where it is able to achieve the aforementioned goals, and is the current gold standard of care for ATAAD. Even in cases where an extended arch repair may be indicated, if a non-aortic surgeon does not have experience with an extended arch repair and it is not feasible, a hemiarch repair is still a reasonable initial life-saving intervention, and distal pathologies may be addressed at a later time if required.

### 3.2. AMDS Hybrid Prosthesis

The AMDS hybrid prosthesis (AMDS) is a more recent development in the realm of ATAAD with utility specifically in Debakey Type I aortic dissection. The AMDS has been demonstrated to be safe in clinical settings and is relatively easy to incorporate into a hemiarch repair for the non-aortic cardiac surgeon [30,31,32]. The most significant benefit has been demonstrated in patients with signs of malperfusion as well as in reductions in the incidence of distal anastomotic new entry tears (DANE) [30,32]. In cases of standard hemiarch repair, DANE has been identified in up to 70% of cases, while in the AMDS, this has been as low as 0% [33]. DANE has been associated with adverse aortic remodeling due to continued FL perfusion following repair [6,32,33]. Following AMDS implantation, 3-year follow-up data have been published from the DARTS trial, which found 30-day mortality of 13.0% and 3-year mortality of 21.7%. There were no cases of device explantation during a 3-year follow-up [30]. Other retrospective studies have supported the findings of safety with the AMDS with rates of in-hospital mortality ranging from 16 to 18% and rates of stroke of 4% [34,35].

While the AMDS is widely available, the experience to date is currently limited compared to other forms of aortic intervention, with select clinical trial or retrospective data available. The DARTS trial was the first clinical trial using the AMDS and included 46 patients. There are ongoing clinical trials and registries that aim to include larger numbers of patients in order to address the limited available data and provide more detailed data on the outcomes of these patients. One such trial is the A ProspEctive, Single ARm, Multi-center Clinical InveStigation to EValuatE the Safety and Effectiveness of AMDS in the TREatment of Acute DeBakey Type I Dissection (PERSEVERE) Trial, which has completed enrollment of 93 patients and is currently in the follow-up phase [7]. The Post-market Registry of the AMDS for the Treatment of Acute DeBakey Type I Dissection (PROTECT) Registry is an international registry collecting information on patients who receive the AMDS and is aiming to enroll 300 patients. The upcoming data from these trials and registries as well as other future studies will help guide the use of the AMDS. Currently, it is being used to facilitate positive aortic remodeling, prevent DANE, and treat malperfusion. Cases such as aortic arch entry tears, head vessel communications, or a more extensive aortic repair being required have been considered contraindications to AMDS use.

### 3.3. Total Arch Repair

Given that aortic arch interventions are diverse as described above, the patients who may require an arch intervention are as well. The goal of an extended arch intervention is to address any distal entry tears or to prevent adverse aortic arch remodeling and so are utilized in Debakey I aortic dissection [4,19,22,23,24,25,36]. In the case of an aortic arch tear or distal entry tears, resection of these tears through a more distal aortic anastomosis or covering the tears with a FET will prevent perfusion of the false lumen and reduce the risk of adverse aortic remodeling [1,19,22,23,24,25]. In the case of an aneurysmal aortic arch, resection of the arch and replacement with a graft or exclusion of the aneurysm with an antegrade TEVAR will prevent further aneurysmal degeneration. FET may also facilitate distal positive remodeling in patients with extensive dissection. Furthermore, patients at increased risk for reintervention, such as those with connective tissue disease or younger patients who will have more time for aneurysmal degeneration, may benefit from a more extensive repair.

One consideration when deciding between a hemiarch and extended arch repair is the increased risk associated with an extended arch repair. Even in the hands of an experienced aortic surgeon, an extended arch repair is a significantly longer and more complex procedure [10,24,36,37,38]. Outcomes following extended arch repairs have improved over recent years and have even become comparable to hemiarch repairs with direct comparisons of in-hospital mortality, including 4% vs. 1%, 3% vs. 3.5%, and 19% vs. 21%, for hemiarch and total arch, respectively, in each comparison [4,19,20,23,24,37,38]. Rates of stroke have generally favored hemiarch repair, with rates ranging from 4.0 to 10.8% for hemiarch and 8.4 to 29.2% for TAR [38,39]. Other studies have demonstrated long-term mortality that is comparable between hemiarch and TAR with >5 year mortality of 7–29.2% and 10.8–30.0%, respectively [10,39,40,41] FunF. Nevertheless, the associated risk with a more extensive procedure cannot be disregarded, especially in higher risk or more frail patients. Furthermore, the risk of spinal cord ischemia with FET deployment and the covering of spinal arteries must be considered with rates of 1.0–5.0% [4,20,23,25,36,38]. Even with comparable outcomes between hemiarch and total arch repairs, it must be remembered that in most cases, total arch repairs in retrospective studies are performed by aortic surgeons, while hemiarch repairs are often performed by a mix of aortic and non-aortic surgeons. The primary surgeon must also consider their abilities and experience with extended arch repairs before proceeding with one of these procedures. In cases where an extended arch repair would be at high risk to the patient, a decision must be made between forgoing the extended arch repair, referring the patient to a high-volume center for the initial intervention, or proceeding with a hemiarch repair and planning for follow-up and potential future interventions on the aortic arch [1].

## 4. Indications and Unanswered Questions

Currently, the answer to which aortic intervention is best in ATAAD is unclear and depends on a host of factors. In cases with an isolated entry tear in the ascending aorta and no other complicating factors, a hemiarch repair is the standard of care. In cases such as this with a Debakey type I aortic dissection, but with signs of malperfusion, the AMDS is a suitable addition to the hemiarch repair for many patients and most cardiac surgeons, even those without formal aortic training.

As mentioned previously, there are several indications for an extended arch repair [1,2], although the decision to proceed with an extended arch repair is not always straightforward. There may be cases where an arch repair is indicated but cannot be performed safely, either due to a lack of surgeon experience with extended arch interventions and without available support from an aortic surgeon or due to prohibitive risk. In these cases, attempting an extended arch repair may result in increased risk to the patient. In these cases, a hemiarch repair may be undertaken in order to address the most immediately life-threatening concern, leaving the aortic arch for another day, where a reintervention on the aortic arch may be performed at a later time at lower risk. If feasible, the indicated procedure should always be performed at the indexed surgery and initial referral to or transfer to a nearby center with experience in aortic surgery may provide a path for extended arch repairs without increasing risk to the patient.

The more conservative approach of a hemiarch repair, even in patients who may benefit from extended arch interventions has been the standard when being performed by non-aortic surgeons. While this may provide the safest short-term risk profile to the patient, it is not without additional risk itself. In patients with distal arch tears or aneurysmal arches, adverse aortic remodeling is known to occur. The primary surgeon at the time of initial ATAAD repair must balance risks and benefits of performing an extended arch repair, considering their comfort with this technique. In cases where an extended arch repair would not be safe, a hemiarch repair is a life-saving procedure and allows for future elective intervention of the arch when required. While these may be addressed at a later stage, aortic reintervention, especially in the aortic arch, carries significant risk both in postoperative outcomes and related to reoperation and a more complex arch repair [5,6]. In cases where there is an indication for an extended arch repair and the surgeon can safely perform this procedure, an extended arch repair should be performed.

As there is insufficient evidence to advocate strongly for one approach over the other in many cases, further investigation into this field is warranted. A key question is whether an extended arch repair in the hands of an aortic surgeon is comparable in short-term risk to a hemiarch repair in the hands of a non-aortic surgeon or an aortic surgeon. If a total arch repair is equivalent in operative risk to a hemiarch repair in the hands of an aortic surgeon, then an extended arch repair may be preferred as an initial strategy. Importantly, if an extended arch repair in the hands of an aortic surgeon is comparable in risk to a hemiarch repair in the hands of a non-aortic surgeon, this may suggest that the availability of aortic surgeons for most, or all, ATAAD cases may be the safest option, as this would provide the ability of the primary surgeon to offer the best surgical intervention for ATAAD patients. This model has been adopted in some centers with a dedicated aortic surgeon on each day for “aortic call”. The ideal approach to addressing these questions is through a randomized control trial, where the standard of care hemiarch repair can be compared to extended arch interventions in order to determine whether a dedicated aortic call and more complex initial intervention is warranted in the case of ATAAD.

## 5. Discussion

ATAAD is a life-threatening condition requiring emergent intervention in order to prevent morbidity and mortality. Various approaches to addressing ATAAD exist, with the hemiarch being the standard of care and the most widely utilized intervention (Table 1). The hemiarch repair offers a relatively safe surgical repair for ATAAD. All cardiac surgeons are taught the hemiarch repair during training, irrespective of formal advanced aortic training, although this procedure can still be challenging in difficult cases. Signs of malperfusion are a significant concern for patients presenting with ATAAD given the association between malperfusion and preoperative and postoperative mortality, morbidity, and required reintervention [42]. Recent advancements in the field of aortic surgery, such as the AMDS hybrid prosthesis, provide the non-aortic cardiac surgeon with a tool to address distal malperfusion and influence positive aortic remodeling. Other approaches have been described for addressing malperfusion, including endovascular repair as the primary intervention in order to correct malperfusion and allow stabilization of the patient prior to surgical repair [43].

Extended arch repairs such as a total arch replacement, ET, or FET have been demonstrated to be safe and effective for addressing various pathologies and improving long-term outcomes in the hands of aortic surgeons. While extended arch repairs may be indicated, they may not be feasible or safe in all circumstances. When indications for extended arch repairs are present, the primary surgeon must decide if it is safe to proceed with extended arch repair, if a hemiarch is sufficient as the initial intervention, or if referral to a high volume center is indicated. There are also cases in which resources may not be available to perform more advanced aortic interventions, such as centers without access to hybrid stents, FET devices, or others. In these cases and where a traditional extended arch repair is not feasible, the hemiarch repair remains the standard of care for ATAAD to reduce the risk of short-term morbidity and mortality. Ultimately, there is no individual gold standard for managing ATAAD but rather several potential strategies that have utility depending on patient presentation and surgeon experience. Until strong evidence is presented for one approach, each case of ATAAD must be evaluated, and the surgical plan must be individualized to the patient. Short-term risk must always be weighed with long-term benefit in order to determine the optimal initial surgical intervention and postoperative course for each patient.

## 6. Future Directions

The question remains regarding which patients may benefit from more extensive arch repairs and how best to provide this care to patients. Further evidence in the form of randomized control trials are necessary in order to determine the optimal intervention for patients presenting with ATAAD and whether formal adjustments to how care is delivered, in the form of dedicated aortic call or referral programs, are warranted to provide optimal care for ATAAD patients. While a hemiarch repair will provide the lowest short-term risk for ATAAD patients, extended arch interventions should be considered where reasonably safe and feasible, and newer technologies such as the AMDS and hybrid FET grafts may help to facilitate more advanced interventions. Additionally, innovative approaches to aortic dissection repair, such as endovascular aortic arch replacements, are currently being developed and may play a role in aortic arch interventions in the future.

## Figures and Tables

**Table 1:** Summary of aortic arch interventions for acute type A aortic dissection.

Aortic Arch Management	Hemiarch Repair	AMDS	Total Arch Repair and FET
Technique	Zone 0, beveled distal anastomosis.	Zone 0 transverse distal anastomosis is most common. Antegrade stent deployment 1 cm proximal to the innominate artery.	Distal anastomosis zone 0–3. Head vessel reconstruction required. Antegrade or retrograde stent deployment for FET. Retrograde deployment can be staged.
Pros	Simplest procedure. Can be performed by any cardiac surgeon. Lowest operative risk. Can be used in all ATAAD.	No significant increase in operative time or complexity. Safe implantation. Can be used by any cardiac surgeon. Reduced risk of DANE. Significant benefits for aortic remodeling and in patients with malperfusion.	Versatile with many approaches. Can address the entire aortic arch. Reduced risk of adverse aortic remodeling. Facilitates future interventions.
Cons	Inability to address more distal disease. Does not address arch disease. Higher risk of adverse remodeling.	Expensive device. Contraindicated in arch tear or aneurysmal arch.	More technically challenging. Higher surgical risk. Increased risk of spinal cord ischemia.
Indications	Standard of care for all ATAAD patients.	Debakey type I aortic dissection. Signs of malperfusion. Prevention of DANE.	ATAAD complicated by arch tears, malperfusion, arch aneurysm, connective tissue disease, young patient, or any presentation not adequately addressed with hemiarch repair.
Contraindications	No absolute contraindications. Aneurysmal aortic arch. Aortic arch tear.	Aneurysmal aortic arch. Aortic arch tear. Head vessel false lumen communications.	Straightforward ATAAD adequately addressed with a hemiarch repair. Limited surgeon experience with arch repair.

FET, frozen elephant trunk; ATAAD, acute type A aortic dissection; DANE, distal anastomotic new entry tear.

## Data Availability

No datasets were created in the production of this manuscript.

## References

[1] Malaisrie S.C., Szeto W.Y., Halas M., Girardi L.N., Coselli J.S., Sundt T.M., Chen E.P., Fischbein M.P., Gleason T.G., Okita Y. (2021). The American Association for Thoracic Surgery expert consensus document: Surgical treatment of acute type A aortic dissection. J. Thorac. Cardiovasc. Surg..

[2] Spanos K., Nana P., von Kodolitsch Y., Behrendt C.-A., Kouvelos G., Panuccio G., Athanasiou T., Matsagkas M., Giannoukas A., Detter C. (2022). Management of Ascending Aorta and Aortic Arch: Similarities and Differences Among Cardiovascular Guidelines. J. Endovasc. Ther..

[3] Sfeir P.M., Issa K., Ayoub C.M. (2021). Mesenteric Malperfusion Syndromes in Type A Aortic Dissection: Current Management Strategies. J. Cardiothorac. Vasc. Anesth..

[4] Rathore K., Newman M. (2022). Aortic Root and Distal Arch Management During Type A Aortic Dissection Repair: Expanding Horizons. Braz. J. Cardiovasc. Surg..

[5] Ergin M., Phillips R.A., Galla J.D., Lansman S.L., Mendelson D.S., Quintana C.S., Griepp R.B. (1994). Significance of distal false lumen after type A dissection repair. Ann. Thorac. Surg..

[6] Tamura K., Chikazawa G., Hiraoka A., Totsugawa T., Sakaguchi T., Yoshitaka H. (2017). The prognostic impact of distal anastomotic new entry after acute type I aortic dissection repair. Eur. J. Cardio-Thorac. Surg..

[7] Szeto W.Y., Fukuhara S., Fleischman F., Sultan I., Brinkman W., Arnaoutakis G., Takayama H., Eudailey K., Brinster D., Jassar A. A novel hybrid prosthesis for open repair of acute DeBakey type I dissection with malperfusion: Early results from the PERSEVERE trial. J. Thorac. Cardiovasc. Surg..

[8] Bayamin K., Power A., Chu M.W.A., Dubois L., Valdis M. (2022). Malperfusion syndrome in acute type A aortic dissection: Thinking beyond the proximal repair. J. Card. Surg..

[9] Yousef S., Brown J.A., Serna-Gallegos D., Navid F., Zhu J., Thoma F.W., Bianco V., Aranda-Michel E., Diaz-Castrillon C.E., Sultan I. (2024). Central versus peripheral cannulation for acute type A aortic dissection. J. Thorac. Cardiovasc. Surg..

[10] Colli A., Carrozzini M., Francescato A., Galuppo M., Comisso M., Toto F., Gregori D., Gerosa G. (2018). Acute DeBakey Type I aortic dissection without intimal tear in the arch: Is total arch replacement the right choice?. Interact. Cardiovasc. Thorac. Surg..

[11] Saran N., Pochettino A. (2021). Cannulation strategies & circulation management in type-A aortic dissection. J. Card. Surg..

[12] Peterss S., Pichlmaier M., Curtis A., Luehr M., Born F., Hagl C. (2017). Patient management in aortic arch surgery. Eur. J. Cardio-Thorac. Surg..

[13] Ramaprabhu K., Saran N., Dearani J., Lahr B., Schaff H., Greason K., Yalamuri S., Mangukia C., Stulak J., Bagameri G. (2022). Cannulation strategies for acute type A dissection-role of central cannulation. Eur. J. Cardio-Thorac. Surg..

[14] Kitamura T., Nie M., Horai T., Miyaji K. (2017). Direct True Lumen Cannulation (“Samurai” Cannulation) for Acute Stanford Type A Aortic Dissection. Ann. Thorac. Surg..

[15] Sosnowski A.W., Jutley R.S., Masala N., Alexiou C., Swanevelder J. (2008). How I do it: Transapical cannulation for acute type-A aortic dissection. J. Cardiothorac. Surg..

[16] Peterson M.D., Garg V., Mazer C.D., Chu M.W.A., Bozinovski J., Dagenais F., MacArthur R.G., Ouzounian M., Quan A., Jüni P. (2022). A randomized trial comparing axillary versus innominate artery cannulation for aortic arch surgery. J. Thorac. Cardiovasc. Surg..

[17] Shelstad R.C., Reeves J.G., Yamanaka K., Reece T.B. (2016). Total Aortic Arch Replacement: Advantages of Varied Techniques. Semin. Cardiothorac. Vasc. Anesth..

[18] Abjigitova D., Veen K.M., van Tussenbroek G., Mokhles M.M., Bekkers J.A., Takkenberg J.J.M., Bogers A.J.J.C. (2022). Cerebral protection in aortic arch surgery: Systematic review and meta-analysis. Interact. Cardiovasc. Thorac. Surg..

[19] Iino K., Takago S., Saito N., Ueda H., Yamamoto Y., Kato H., Kimura K., Takemura H. (2022). Total arch replacement and frozen elephant trunk for acute type A aortic dissection. J. Thorac. Cardiovasc. Surg..

[20] Ho J.Y.K., Chow S.C.Y., Kwok M.W.T., Fujikawa T., Wong R.H.L. (2021). Total Aortic Arch Replacement and Frozen Elephant Trunk. Semin. Thorac. Cardiovasc. Surg..

[21] Ibrahim M., Stevens L.-M., Ouzounian M., Hage A., Dagenais F., Peterson M., El-Hamamsy I., Boodhwani M., Bozinovski J., Moon M.C. (2021). Evolving Surgical Techniques and Improving Outcomes for Aortic Arch Surgery in Canada. CJC Open.

[22] Chu M.W., Losenno K.L., Dubois L.A., Jones P.M., Ouzounian M., Whitlock R., Dagenais F., Boodhwani M., Bhatnagar G., Poostizadeh A. (2019). Early Clinical Outcomes of Hybrid Arch Frozen Elephant Trunk Repair With the Thoraflex Hybrid Graft. Ann. Thorac. Surg..

[23] Chung J.C.-Y., Ouzounian M., Chu M.W.A., El-Hamamsy I. (2020). The Evolving Role of Hybrid Arch Repair. Innov. Technol. Tech. Cardiothorac. Vasc. Surg..

[24] Hayashi J., Nakajima H., Asakura T., Sho R., Tokunaga C., Takazawa A., Yoshitake A. (2022). Safety and arch complications after hemiarch versus total arch replacement with stented elephant trunk in acute type 1 dissection: Is a stent graft always beneficial?. JTCVS Open.

[25] Hage A., Hage F., Dagenais F., Ouzounian M., Chung J., El-Hamamsy I., Peterson M.D., Boodhwani M., Bozinovski J., Moon M.C. (2022). Frozen Elephant Trunk for Aortic Arch Reconstruction is Associated with Reduced Mortality as Compared to Conventional Techniques. Semin. Thorac. Cardiovasc. Surg..

[26] Furutachi A., Takamatsu M., Nogami E., Hamada K., Yunoki J., Itoh M., Kamohara K. (2019). Early and mid-term outcomes of total arch replacement with the frozen elephant trunk technique for type A acute aortic dissection. Interact. Cardiovasc. Thorac. Surg..

[27] Rorris F.P., Antonopoulos C.N., Gissis I., Tsagakis K., Kokotsakis J. (2022). E-Vita OPEN NEO Hybrid Stent Graft: A New Frontier for Total Arch Replacement. Ann. Vasc. Surg..

[28] Ahmad A.E.-S., Silaschi M., Borger M., Seidiramool V., Hamiko M., Leontyev S., Zierer A., Doss M., Etz C.D., Benedikt P. (2023). The Frozen Elephant Technique Using a Novel Hybrid Prosthesis for Extensive Aortic Arch Disease: A Multicentre Study. Adv. Ther..

[29] Roselli E.E., Vargo P.R., Bakaeen F., Koprivanac M., Burns D., Kuramochi Y., Gillinov M., Soltesz E., Tong M., Unai S. (2023). Branched stented anastomosis frozen elephant trunk repair: Early results from a physician-sponsored investigational device exemption study. J. Thorac. Cardiovasc. Surg..

[30] Bozso S.J., Nagendran J., Chu M.W.A., Kiaii B., El-Hamamsy I., Ouzounian M., Forcillo J., Kempfert J., Starck C., Moon M.C. (2024). Three-year outcomes of the Dissected Aorta Repair Through Stent Implantation trial. J. Thorac. Cardiovasc. Surg..

[31] Bozso S.J., Nagendran J., MacArthur R.G.G., Chu M.W.A., Kiaii B., El-Hamamsy I., Cartier R., Shahriari A., Moon M.C. (2019). Dissected Aorta Repair Through Stent Implantation trial: Canadian results. J. Thorac. Cardiovasc. Surg..

[32] Bozso S.J., Nagendran J., Chu M.W., Kiaii B., El-Hamamsy I., Ouzounian M., Kempfert J., Starck C., Moon M.C. (2021). Midterm Outcomes of the Dissected Aorta Repair Through Stent Implantation Trial. Ann. Thorac. Surg..

[33] Rylski B., Hahn N., Beyersdorf F., Kondov S., Wolkewitz M., Blanke P., Plonek T., Czerny M., Siepe M. (2017). Fate of the dissected aortic arch after ascending replacement in type A aortic dissection. Eur. J. Cardio-Thorac. Surg..

[34] Luehr M., Gaisendrees C., Yilmaz A.K., Winderl L., Schlachtenberger G., Van Linden A., Wahlers T., Walther T., Holubec T. (2023). Treatment of acute type A aortic dissection with the Ascyrus Medical Dissection Stent in a consecutive series of 57 cases. Eur. J. Cardio-Thorac. Surg..

[35] Montagner M., Kofler M., Seeber F., Pitts L., Starck C., Sündermann S.H., Kurz S., Grubitzsch H., Falk V., Kempfert J. (2022). The arch remodelling stent for DeBakey I acute aortic dissection: Experience with 100 implantations. Eur. J. Cardio-Thorac. Surg..

[36] Hanif H., Dubois L., Ouzounian M., Peterson M.D., El-Hamamsy I., Dagenais F., Hassan A., Chu M.W. (2018). Aortic Arch Reconstructive Surgery With Conventional Techniques vs Frozen Elephant Trunk: A Systematic Review and Meta-Analysis. Can. J. Cardiol..

[37] Elbatarny M., Stevens L.-M., Dagenais F., Peterson M.D., Vervoort D., El-Hamamsy I., Moon M., Al-Atassi T., Chung J., Boodhwani M. (2024). Hemiarch versus extended arch repair for acute type A dissection: Results from a multicenter national registry. J. Thorac. Cardiovasc. Surg..

[38] Chen J.F., Ouzounian M., Peterson M., Tatangelo M., Dagenais F., Hage A., Lindsay T.F., Chu M.W., Chung J.C. (2024). Outcomes of Total Aortic Arch Replacement in a Canadian Nationwide Registry. Can. J. Cardiol..

[39] Fukunaga N., Wakami T., Shimoji A., Maeda T., Mori O., Yoshizawa K., Tamura N. (2024). Outcomes of ascending aorta and partial arch replacement with entry resection for DeBakey type I acute aortic dissection. Gen. Thorac. Cardiovasc. Surg..

[40] Katayama A., Uchida N., Katayama K., Arakawa M., Sueda T. (2014). The frozen elephant trunk technique for acute type A aortic dissection: Results from 15 years of experience. Eur. J. Cardio-Thorac. Surg..

[41] Ikeno Y., Yokawa K., Koda Y., Gotake Y., Henmi S., Nakai H., Yamanaka K., Inoue T., Tanaka H., Okita Y. (2019). The fate of the downstream aorta after open aortic repair for acute DeBakey type i aortic dissection: Total arch replacement with elephant trunk technique versus non-total arch replacement. Eur. J. Cardio-Thorac. Surg..

[42] Jaffar-Karballai M., Tran T.T., Oremakinde O., Zafar S., Harky A. (2021). Malperfusion in Acute Type A Aortic Dissection: Management Strategies. Vasc. Endovasc. Surg..

[43] Norton E.L., Orelaru F., Naeem A., Wu X., Kim K.M., Williams D.M., Fukuhara S., Patel H.J., Deeb G.M., Yang B. (2022). Treating lower extremity malperfusion syndrome in acute type A aortic dissection with endovascular revascularization followed by delayed aortic repair. JTCVS Open.

